# HNRNPC as a pan-cancer biomarker and therapeutic target involved in tumor progression and immune regulation

**DOI:** 10.32604/or.2024.055866

**Published:** 2024-12-20

**Authors:** YUEZHOU ZHANG, ZHAO ZHANG, JINXIN DONG, CHANGAN LIU

**Affiliations:** 1 Department of Hepatobiliary Surgery, The Second Affiliated Hospital of Chongqing Medical University, Chongqing, 400016, China; 2 Department of Hepatobiliary Surgery, The First Affiliated Hospital of Chongqing Medical University, Chongqing, 400016, China

**Keywords:** Heterogeneous nuclear ribonucleoprotein C (HNRNPC), Pan-cancer analysis, Tumor immunity, Prognostic biomarker, Immunotherapeutic target, Hepatocellular carcinoma

## Abstract

**Background:**

Aberrant expression of RNA-binding proteins (RBPs) has been linked to a variety of diseases, including hematological disorders, cardiovascular diseases, and multiple types of cancer. Heterogeneous nuclear ribonucleoprotein C (HNRNPC), a member belonging to the heterogeneous nuclear ribonucleoprotein (hnRNP) family, plays a pivotal role in nucleic acid metabolism. Previous studies have underscored the significance of HNRNPC in tumorigenesis; however, its specific role in malignant tumor progression remains inadequately characterized.

**Methods:**

We leveraged publicly available databases, including The Cancer Genome Atlas (TCGA), to explore the potential involvement of HNRNPC across various cancers. Additionally, we performed experimental validation studies focused on liver cancer.

**Results:**

Our analysis revealed that HNRNPC is overexpressed in a wide range of common malignancies, including liver and lung cancers, and is strongly linked to unfavorable outcomes. Furthermore, HNRNPC was observed to be closely linked to tumor immunity. Through immune checkpoint analysis and immune cell infiltration assessment, HNRNPC emerged as a potential target for modulating tumor immunotherapy. Notably, silencing of HNRNPC markedly inhibited the proliferation, metastasis, and infiltration of liver cancer cells.

**Conclusion:**

In summary, our findings highlight HNRNPC as a prognostic marker in various cancers, including liver cancer, and suggest its involvement in shaping the tumor immune microenvironment. These insights offer potential avenues for improving clinical outcomes in tumors with elevated HNRNPC expression, particularly through immunotherapeutic strategies.

## Introduction

Cancer remains one of the most formidable public health challenges worldwide, contributing significantly to global mortality [1]. Despite significant research efforts that have effectively reduced overall mortality rates, the biological characteristics of tumors remain poorly understood. Consequently, the underlying mechanisms of tumor development are not yet fully elucidated, which contributes to the persistently high incidence rates [2–4]. As a result, enthusiasm for cancer research remains strong, with ongoing efforts to unravel the complexities of this disease [5,6].

As is well known, genetic mutations and epigenetic variations in tumor proliferation leads to unrestricted tumor growth, immune evasion, and unique metabolic characteristics [7–9]. RNA editing, serving as an important epigenetic mechanism of cellular regulation, exerts an impact on tumor proliferation and differentiation comparable to that of genomic mutations [10]. RNA-binding proteins, which primarily regulate RNA abundance and function post-transcriptionally, play a crucial role in RNA editing and are vital regulators of cellular homeostasis [11]. Among these proteins, heterogeneous nuclear ribonucleoprotein C (HNRNPC), which belongs to the HNRNP family, regulates various facets of RNA processing, such as alternative splicing, mRNA stability, and translation control [12–14]. These findings indicate that HNRNPC might contribute to maintaining tumor cell homeostasis. Given its significant role in RNA editing, HNRNPC could be considered a potential therapeutic target for cancer therapy [15]. Previous evidence has also indicated that abnormal expression of HNRNPC can lead to splicing errors in post-transcriptional modifications, resulting in tumorigenesis [16]. Research has identified the close relationship between HNRNPC and pathways including Signal Transducer and Activator of Transcription 3 (STAT3) and β-catenin, and discoidin domain receptors 1 (DDR1), which play roles in multiple tumors [17–19]. Moreover, some studies suggest that HNRNPC might induce DNA damage, contributing to tumorigenesis through mechanisms that are yet to be fully understood [20]. Despite extensive research on HNRNPC in specific tumors, comprehensive investigations across the entire cancer spectrum remain lacking. Analyzing HNRNPC across various cancers using databases can provide valuable insights into its molecular mechanisms and its predictive value in cancer biology, prognosis, and treatment [11,21–23].

In this research, we performed an extensive analysis of HNRNPC using databases including TCGA and Genotype-Tissue Expression (GTEx), exploring its significance in cancer prognosis. Moreover, some studies have suggested that HNRNPC may influence tumor immunity. For example, research has shown that HNRNPC inhibits the tumor immune microenvironment through the activation of Treg cells, thereby promoting prostate cancer progression. Another study revealed that HNRNPC promotes immune evasion in breast cancer by activating the VIRMA-mediated TFAP2A/DDR1 axis [19,24]. After evaluating the association between HNRNPC and immune checkpoint activity and immune cell infiltration, we identified that HNRNPC has an indispensable relationship with tumor immunity in many common cancers. These findings may provide new insights into tumor immunotherapy. We also investigated the relationship between tumor mutation burden (TMB), tumor stemness score, microsatellite instability (MSI), and HNRNPC to gain a more comprehensive understanding of its role in cancer treatment.

## Materials and Methods

### Acquiring comprehensive pan-cancer data

We obtained comprehensive datasets from the Genotype-Tissue Expression (GTEx) repository (https://www.gtexportal.org) (accessed on 22 October 2024) and The Cancer Genome Atlas (TCGA) program (https://portal.gdc.cancer.gov/) (accessed on 22 October 2024), accessed through the UCSC database (https://xenabrowser.net/) (accessed on 22 October 2024) [25].

These datasets include 10,534 samples from TCGA and 15,776 samples from GTEx. We filtered out samples with zero expression levels and log2-transformed expression values where applicable, particularly when analyzing differential expression. Following the exclusion of cancer types with fewer than three cases as well as those with follow-up durations of less than 30 days, we obtained mRNA sequencing data for 34 different cancer types. These data were accompanied by related clinical attributes, including patient demographics, survival status, clinical presentations, and pathological staging. Comparative analysis of HNRNPC expression levels was performed using R packages, utilizing “limma”, “ggplot2”, and “ggpubr” (version 3.6.3, https://www.R-project.org) (accessed on 22 October 2024) [26–28].

### Pan-cancer prognosis assessment

For comprehensive pan-cancer prognosis assessment, we refined the data by excluding samples with no expression and those with a survival period of less than 30 days. This ensured high-quality prognostic expression data to ensure robust analyses. We then utilized the Cox proportional hazards regression model with the “Coxph” R package (version 3.6.3) to investigate the relationship between gene levels and prognosis across diverse cancer types [29]. Prognostic outcomes were rigorously assessed using the Logrank test, subsequently creating Kaplan-Meier survival curves for each cancer subtype. The prognostic significance of HNRNPC was evaluated across various cancer types, focusing on four key clinical endpoints: disease-free survival (DFS), overall survival (OS), disease-specific survival (DSS), and progression-free survival (PFS). 95% confidence intervals, hazard ratios (HR), and *p*-values indicative of statistical significance were calculated, with *p* < 0.05 considered significant.

### Investigating the correspondence between HNRNPC and clinical manifestations

We performed an extensive analysis of the relationship between HNRNPC expression levels and distinct clinical stages within each tumor type utilizing the R packages “ggpubr” and “limma” (version 3.6.3). ROC-AUC values for HNRNPC expression at a pan-cancer level were calculated using the R packages “pROC” and “ggplot2”. This assessment offered valuable perspectives on the predictive potential of HNRNPC across diverse types of cancer, elucidating its diagnostic efficacy and utility in clinical settings.

### Correlation analysis of immune characteristics

First, utilizing a standardized dataset (https://portal.gdc.cancer.gov/) encompassing various cancers, we examined the expression profiles of HNRNPC in conjunction with 150 reference genes related to immune pathway, including 18 chemokine receptors, 41 chemokines, 24 immunoinhibitors, 21 MHC-related genes, and 46 immunostimulators, utilizing the standardized dataset encompassing various cancers. Subsequently, we retrieved HNRNPC gene expression data along with 60 immune checkpoint markers, 18 chemokine receptors, 41 chemokines, and 21 MHC-related immune pathway markers from the comprehensive dataset [30]. We excluded samples with zero expression levels as well as all control samples from the analysis. Furthermore, each expression value underwent a log_2_(x + 0.001) conversion.

Subsequently, Pearson’s correlation coefficients between HNRNPC and the indicator genes associated with the five immune pathways were computed utilizing the “limma” software in R (version 3.6.3). The “ESTIMATE” package in R (version 3.6.3) was employed to compute the StromalScore, ImmuneScore, and ESTIMATE scores for 10,180 tumor specimens across 44 different cancer categories [31]. We evaluated the correlation coefficient between HNRNPC and immune infiltration scores in each tumor using the corr.test function from the psych package in R to identify immune infiltration scores that were significantly correlated with statistical significance. To evaluate T cell CD4, macrophage, B cell, neutrophil, T cell CD8, and dendritic cell infiltration levels per patient across each tumor, we utilized the Timer method, mcpcounter method, deconvo_quantiseq method, and deconvo_epic method from the IOBR package in R (version 3.6.3). For the visualization of results, we employed the reshape2 and RColorBrewer packages in R (version 3.6.3) [32,33].

### The association between HNRNPC and genome heterozygosity and stemness

Current research suggests that the genomic heterogeneity of tumors has a significant impact on the effectiveness of immune checkpoint inhibitor (ICI) therapy and patient prognosis, including indicators such as microsatellite instability (MSI), tumor purity, and tumor mutation burden (TMB) [34]. We obtained the dataset of all TCGA specimens analyzed by the MuTect2. Utilizing the “tmb” function from the maftools package (version 3.6.3, https://www.R-project.org), we computed the TMB for every individual tumor. Subsequently, we merged the TMB data with transcriptomic data and applied a log_2_(x + 0.001) conversion to every expression measurement. Finally, we evaluated the relationship between TMB and gene expression.

Malta et al. provided six measures of tumor stemness based on mRNA expression and methylation signatures, namely RNAss, DNAss, ENHss, DMPss, EREG, EXPss, and EREG-METHss [35]. Utilizing the OCLR algorithm provided by them, we obtained tumor stemness indices for different malignancies. Following this, we combined the stemness metrics with gene expression information, inferred the Pearson correlation between them, and visualized the results.

#### Cell lines and cultures

The liver cancer cell lines HCC-LM3 and HuH-7 were obtained from the Type Culture Collection at the Chinese Academy of Sciences. These cell cultures were meticulously maintained under ideal conditions at 37°C with a 5% carbon dioxide (CO_2_) concentration. The culture medium employed for their growth comprised DMEM (Dulbecco’s Modified Eagle Medium) (Gibco, Waltham, MA, USA, CAT: 11965092) obtained from Corning Incorporated. This culture was enriched with 10% fetal bovine serum (Gibco, High Point, NC, USA, CAT: A3160902) and 1% penicillin/streptomycin (Gibco, High Point, NC, USA, CAT: 15140148) to provide essential nutrients and maintain sterility throughout the culture period.

#### Cell transfection

To examine the functional role of HNRNPC, we utilized specific short hairpin RNAs (shRNAs) targeting HNRNPC, along with a scrambled shRNA control, synthesized by GenePharma. Cultured cells were plated with 8 × 10^5^ cells per chamber in 6-well plates to ensure optimal growth conditions. Transfection procedures were performed when cells reached approximately 70% confluence, using Lipofectamine 3000 reagent (Invitrogen, San Diego, CA, USA, CAT: L3000150) according to the manufacturer’s instructions. Briefly, 1 mL DMEM containing the virus stock solution (1 × 10^8^ TU/mL) and transfection reagent was added to each well, with a multiplicity of infection (MOI) of 10, 5, and 1 for the lentivirus targeting HNRNPC (shRNA, GenePharma, HNRNPC sense 5′-GGCCTCTATGACTCGCATGT-3′, antisense 5′-TTTGTAGGACGGGGTGTAGC-3′) and the empty vector lentivirus (control). After 6–10 h, when a small number of floating cells were observed under the microscope (CX31, Olympus, Tokyo, Japan), the medium was replaced with DMEM supplementary with 10% FBS. This transfection protocol facilitated efficient delivery of the shRNAs into the cells, enabling specific modulation of HNRNPC expression levels for subsequent functional analyses.

#### RNA isolation and quantitative real-time PCR (qRT-PCR)

Gene expression was quantified using a two-step qRT-PCR method. Total RNA was isolated from both cellular and subcutaneous tumor tissues of mice using TRIzol. (Takara Osaka, Japan CAT: 9108) The subsequent conversion of RNA to first-strand complementary DNA (cDNA) was achieved using a cDNA synthesis kit (MCE, South Plainfield, NJ, USA, CAT: HY-K0512). The expression of various transcripts was assessed by real-time PCR amplification 95°C for 5 min; (95°C for 10 s, 60°C for 30 s; 40 cycles); 95°C for 15 s; 60°C for 1 min; 95°C for 15 s with SYBR Green (MCE, South Plainfield, NJ, USA CAT: HY-K0501), using the CFX96-Real-Time System (Bio-Rad, Hercules, CA, USA) with GAPDH serving as the internal reference gene. The primer sequences used for amplification targeted HNRNPC (sense: 5′-GGCCTCTATGACTCGCATGT-3′; antisense: 5′-TTTGTAGGACGGGGTGTAGC-3′) and GAPDH (sense: 5′-TGTTGCCATCAATGACCCCTT-3′; antisense: 5′-CTCCACGACGTACTCAGCG-3′). GAPDH was chosen as the internal reference gene due to its stable expression across different experimental conditions, Calculate the relative expression of mRNA using the 2^−ΔΔCt^ method.

#### Cell counting Kit-8 (CCK-8) analyses

Cell viability in HCC cells was assessed using the CCK-8 reagent (Boster Biological Technology, Wuhan, China, CAT: AR1160-100) following the manufacturer’s instructions. Cells were plated at a density of 2 × 10^4^ cells per well in a 96-well plate, and absorbance measurements were taken at 450 nm with a microplate reader at 12, 24, 48, and 72 h.

#### Colony formation assay

The Colony Formation Assay was used to assess the clonogenic capability of hepatocellular carcinoma (HCC) cells. Cells were seeded at a density of 400 cells per well in a 6-well plate and cultured in DMEM supplemented with 10% FBS at a temperature of 37°C. After 15 days, colonies were stained with crystal violet (Beyotime, Shanghai, China, CAT: C0121) and counted to evaluate the colony formation efficiency. The 15-day incubation period was selected to allow for the formation of visible colonies, ensuring that the assay could accurately reflect the clonogenic potential of the cells. The colony formation rate was calculated as follows: (number of colonies/numbers of seeded cells) × 100%.

#### Western blot

Cell lysates were prepared using lysis buffer (Invitrogen, San Diego, CA, USA CAT: FNN0021), and the same amount (20 μL) of total protein was loaded into SDS-PAGE gels for electrophoresis. Subsequently, separated proteins were electrotransferred at 120 volts onto PVDF membranes (Millipore, Burlington, MA, USA, CAT: IPVH00010). The membranes were blocked with Protein Free Blocking Buffer (Seville, Wuhan, China, CAT: G2052) for 20 min, followed by overnight incubation at 4°C with primary antibodies diluted to 1:1000 (GAPDH and HNRNPC antibodies from PROTEINTECH, Chicago, USA GAPDH CAT: 10494-1-AP; HNRNPC CAT: 68447-1-Ig; B-catenin and p-STAT3 antibodies from Cell Signaling Technology, Boston, MA, USA, B-catenin CAT: 9582S, p-STAT3 CAT: 9145T). After that, the membranes were incubated with HRP-conjugated secondary antibodies (Seville, Wuhan, China, CAT: GB23303 1:500) at room temperature for 2 h. Finally, detection was performed using a chemiluminescence enhancement kit (Beyotime, Shanghai, China, CAT: P0018S) on a ChemiDoc Imaging system (BIO-RAD Hercules, CA, USA).

#### Wound scratch assay

Cells were seeded in a 6-well plate and allowed to form a confluent monolayer overnight. Following serum starvation for 24 h, a scratch was induced through 10 μL pipette tip scratching, followed by three washes with 10 × PBS. The initial wound width was measured at *t* = 0, and migration was quantified as the percentage of gap closure at *t* = 48 h relative to *t* = 0. All experiments were conducted in triplicate.

#### Cell migration and invasion assay

The cell migration and invasion assay were performed using a 24-well Boyden chamber fitted with an 8 μm pore size polycarbonate membrane (Corning, New York, NY, USA) to assess the migratory and invasive abilities of the cells. We introduced 200 μL of serum-depleted medium (DMEM) containing 1 × 10^5^ cells into the upper chamber, while the lower chamber was filled with 600 μL of DMEM enriched with 10% FBS, setting up three replicate wells. After incubating at 37°C for 24 h, the wells were washed with PBS, and 4% paraformaldehyde was added to fix the cells remaining on the membrane. The cells were then stained with crystal violet and counted using a light microscope (CX31, Olympus, Tokyo, Japan).

#### Nude mice orthotopic model study

Throughout the entire experimental procedure, strict compliance with animal research ethics was upheld, and approval was obtained from the Institutional Animal Care and Use Committee. (IACUC-CQMU-2023-0439). A cell line-derived xenograft (CDX) model was established using 4-week-old male BALB/c nude mice (Slaike Jinda, Changsha, China). The mice were randomly divided into two groups (5 mice per group) and housed under barrier conditions in Chongqing Medical University Animal Experiment Center (SPF). After anesthesia, a suspension of NC-HCCLM3 cells or SH-HCCLM3 cells (5 × 10^6^) was inoculated subcutaneously into the axillary region of each nude mouse. The volume of the implanted tumors was measured and recorded at 10, 15, 20, 25, and 30 days. After 4 weeks, the mice were euthanized using isoflurane, and xenograft tumor tissues were harvested for further pathological examination and analysis. This study fully complies with the ARRIVE guidelines [36].

### Immunohistochemistry (IHC) staining

IHC visuals depicting HNRNPC protein levels in both cancer and normal tissues of hepatocellular carcinoma were downloaded from HPA (The Human Protein Atlas, https://www.proteinatlas.org) (accessed on 22 October 2024). The Human Protein Atlas (HPA) database provides a comprehensive collection of protein expression patterns across diverse human tissues and cancers. In our study, these images were utilized to assess HNRNPC protein abundance in normal and tumor tissues, contributing to our understanding of its role in liver hepatocellular carcinoma.

#### Immunofluorescence (IF)

Subcutaneous tumor tissue from mice was fixed in 4% paraformaldehyde (Biosharp, Hefei, China, CAT: BL539A), dehydrated, embedded in paraffin, next Using a paraffin microtome (RWD, Shenzhen, China) for sectioning at a thickness of 5 µm. After deparaffinization, antigen retrieval was performed using citrate buffer (Seville, Wuhan, China, CAT: G1202) at 95°C. Next, Triton X-100 was used for permeabilization (Solaibao, Beijing, China, CAT: T8200, 1:400), followed by blocking with goat serum (Boster Biological Technology, Wuhan, China, CAT: AR0009). Primary antibody HNRNPC (PROTEINTECH, Chicago, MI, USA, CAT: 68447-1-Ig,1:200); KI67, (Seville, Wuhan, China, CAT: GB111141-100, 1:500); α-SMA (Seville, Wuhan, China, CAT: GB12044-100, 1:200); Vimentin, (Seville, Wuhan, China, CAT: GB11192-100, 1:200); CD3 (Seville, Wuhan, CAT: GB13014-50, 1:200); CD4, (Seville, Wuhan, China, CAT: GB15064-100,1:200); CD133, (Seville, Wuhan, China, CAT: GB113807-100 1;400); E-cadherin, (Cell Signaling Technology, Boston, MA, USA, CAT: 9145T 1:500); F4/80, (Seville, Wuhan, China, CAT: GB113373-100, 1:200) was incubated overnight at 4°C, followed by incubation with fluorophore-conjugated secondary antibody (Seville, Wuhan, China, CAT: GB21303; GB22301 1:200) at room temperature for 2 h. Nuclei were counterstained with DAPI (Beyotime, Shanghai, China, CAT: P0131), and slides were covered with an cover glass. Immunofluorescence signals were visualized using an Olympus fluorescence microscope (BX63, Olympus, Tokyo, Japan).

### Statistical analysis

Gene expression data from the TCGA and GTEx databases were evaluated using Student’s *t*-test conducted in R software. Patient survival data were analyzed using Kaplan–Meier curves, log-rank tests, and Cox proportional hazards regression models, also performed in R software. Statistical analyses for the experimental part were conducted using GraphPad Prism software (San Diego, CA, USA, version: 10.30), with data presented as mean ± standard deviation (SD), unless otherwise specified, for *in vivo* experiments, the sample size for the knockdown group was n = 5, and the sample size for the control group was n = 5. For normally distributed data, one-way ANOVA was used; for non-normally distributed data, rank correlation tests were employed. The choice between parametric or non-parametric tests was based on the data distribution to ensure appropriate statistical analysis. Statistical significance was defined as two-sided *p* < 0.05, with significance levels indicated as follows: *p < 0.05; **p < 0.01; ***p < 0.001.

## Results

### The HNRNPC expression across multiple cancer types

**Figure 1:**
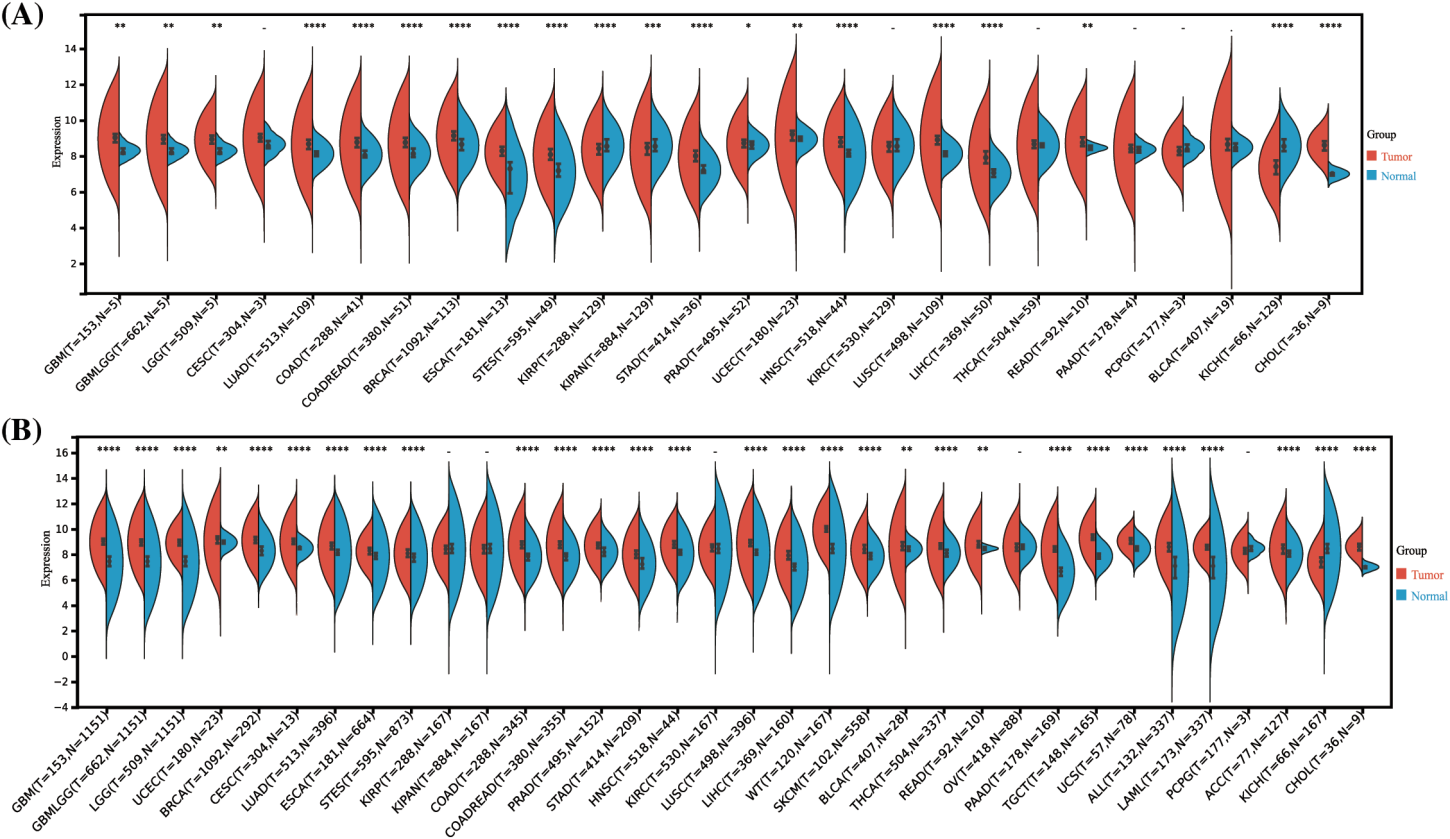
Differential HNRNPC expression across pan-cancer. (A) Assessment of HNRNPC levels in paired cancer and normal tissues utilizing the TCGA database. (B) Comparison of HNRNPC expression variances across TCGA and GTEx repositories. *p < 0.05; **p < 0.01; ***p < 0.001; ****p < 0.0001.

By analyzing data from the TCGA and GTEx repositories, we assessed the transcriptome sequencing profiles of HNRNPC across multiple cancer types, offering insights into its Pan-cancer expression profiling indicated a notable overexpression of HNRNPC in 20 out of 26 prevalent malignancies including breast cancer (BRCA), Liver hepatocellular carcinoma (LIHC), and lung adenocarcinoma (LUAD) (Fig. 1A). Furthermore, with an expanded sample size, HNRNPC exhibited significantly elevated expression across nearly all cancer lineages, with the exceptions of kidney renal papillary cell carcinoma (KIRP), kidney invasive papillary renal cell carcinoma (KIPAN), kidney renal clear cell carcinoma (KIRC), ovarian cancer (OV), and pheochromocytoma and paraganglioma (PCPG) (Fig. 1B). Notably, the most pronounced overexpression was observed in several cancers, including Glioblastoma Multiforme (GBM) (*p* = 1.7e-257), BRCA (*p* = 1.2e-102), Lung Squamous Cell Carcinoma (LUSC) (*p* = 8.2e-108), and LIHC (*p* = 3.3e-49).

### Survival analysis

**Figure 2:**
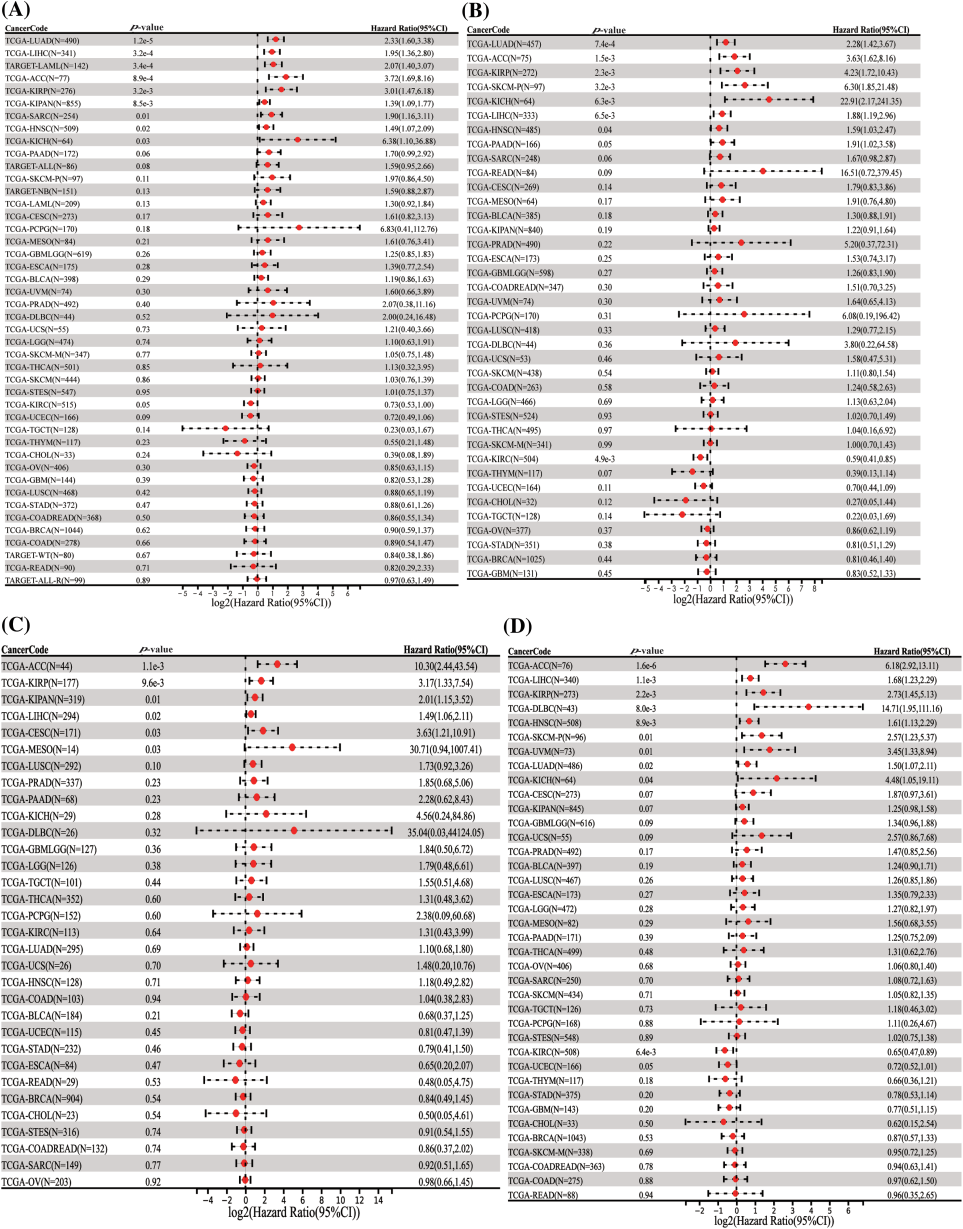
Univariate Cox regression comparison of HNRNPC with OS, DFS, PFS, and DSS in pan-cancer. (A) Association between HNRNPC level and OS; (B) DSS; (C) DFS; (D) PFS. OS represents overall survival; DSS represents disease-specific survival; DFS represents disease-free survival; and PFS represents progression-free survival.

In our comprehensive analysis of survival outcomes across 11 cancer types, we evaluated the influence of HNRNPC expression on disease-specific survival (DSS), overall survival (OS), progression-free survival (PFS), and disease-free survival (DFS). Cox regression analysis demonstrated that higher HNRNPC expression was significantly associated with reduced overall survival (OS) in all 11 cancer types. (Fig. 2A). Specifically, in eight cancer types—LUAD, KIRP, Head and Neck Squamous Cell Carcinoma (HNSC), LIHC, Skin Cutaneous Melanoma (SKCM), Pancreatic Adenocarcinoma (PAAD), Adrenal Cortical Carcinoma (ACC), and Kidney Chromophobe (KICH)—elevated HNRNPC levels were associated with decreased disease-specific survival (DSS) (Fig. 2B). Subsequent analyses suggested a potential correlation between elevated HNRNPC expression and reduced disease-free survival (DFS) in ACC, KIRP, KIPAN, LIHC, Cervical Squamous Cell Carcinoma (CESC), and Mesothelioma (MESO) patients (Fig. 2C). Additionally, our Cox regression analysis demonstrated a robust correlation exists between elevated HNRNPC expression and unfavorable progression-free survival (PFS) in ACC, LIHC, KIRP, Diffuse Large B-Cell Lymphoma (DLBC), HNSC, SKCM, Uveal Melanoma (UVM), LUAD, and KICH (Fig. 2D). These associations were further validated using Kaplan–Meier survival analysis (Fig. S1). Hazard ratios (HR) along with their corresponding 95% confidence intervals were computed to assess the strength of these relationships.

### The correlation between HNPNPC and demographic characteristics

**Figure 3:**
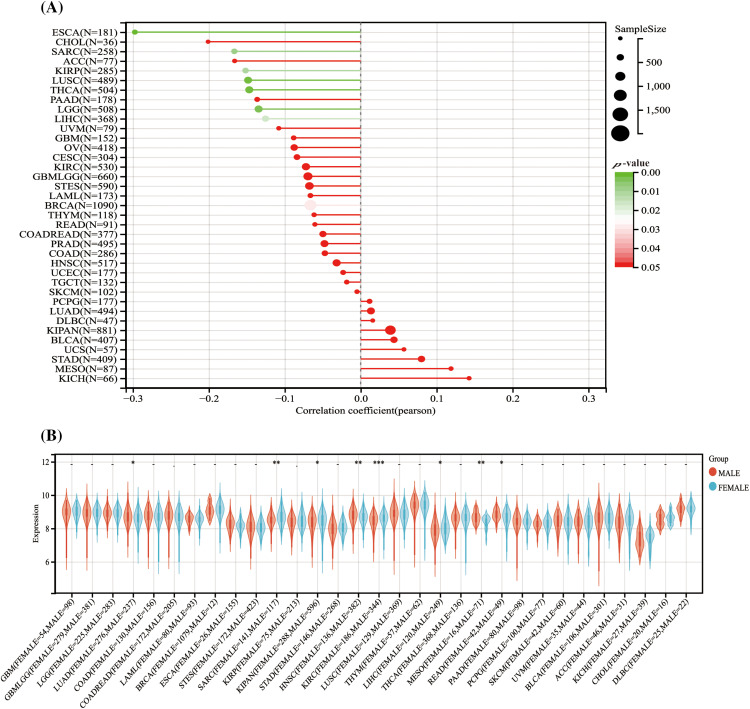
The association between HNRNPC level and the demographic characteristics of individuals diagnosed with cancer. (A) The association between HNRNPC and the age of cancer patients across various cancer types. (B) The association between HNRNPC and the sex of cancer patients across multiple cancer types. *p < 0.05; **p < 0.01; ***p < 0.001.

Analysis of HNRNPC expression levels across different demographic characteristics revealed notable correlations. Among 28 tumor types, including LUSC, THCA, and LIHC, higher HNRNPC expression levels were associated with younger age groups, whereas in 9 tumor types, including LUSC, PAAD, and BRCA, higher expression levels were observed in older age groups (Fig. 3A).

Furthermore, an examination of the relationship between HNRNPC expression and sex indicated distinct patterns. Male patients showed elevated HNRNPC expression levels in LUAD, HNSC, MESO, and Rectum adenocarcinoma (READ), whereas female patients exhibited high expression levels in Sarcoma (SARC), KIPAN, KIRC, and LIHC. However, in the remaining tumor types, no notable correlation was detected between HNRNPC expression and sex (Fig. 3B).

### Relationship of HNRNPC expression to tumor stage, grade, and diagnostic accuracy

**Figure 4:**
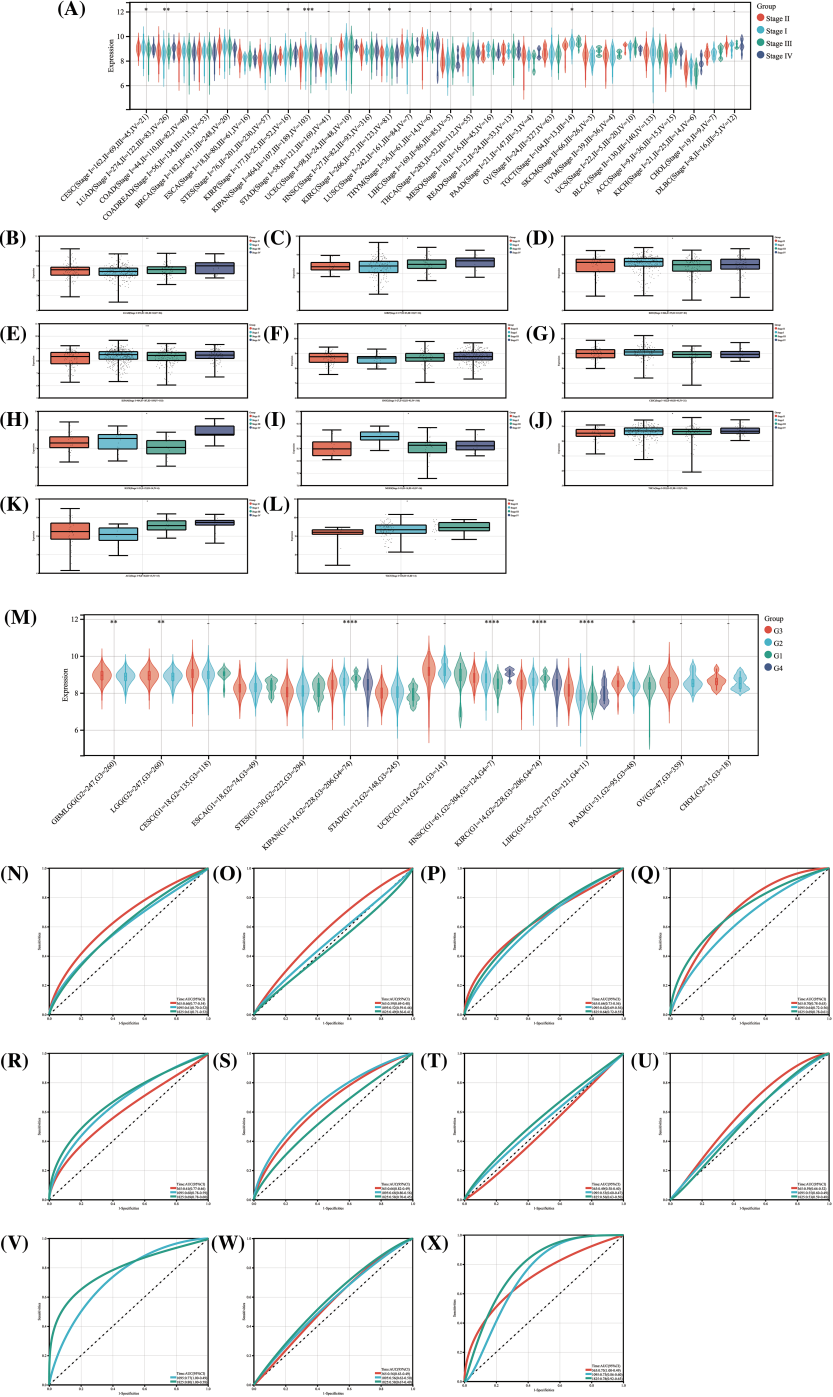
The relationship between HNRNPC and cancer staging and grading, and its diagnostic value. (A) The levels of HNRNPC expression across various stages of cancer. (B–L) HNRNPC expression in different stages of LUAD, KIRP, KIRC, KIPAN, HNSC, CESC, KICH, MESC, THCA, ACC, TGCT. (M) The relationship between different grades of cancer and HNRNPC expression in all cancers. (N–X) ROC curve of HNRNPC in all cancers. *p < 0.05; **p < 0.01; ****p < 0.0001.

Upon assessing HNRNPC expression levels across various clinical and pathological stages in 11 tumor types, significant differences were observed (Fig. 4A). These differences are illustrated in Fig. 4B–L. Notably, in Thyroid Cancer (THCA), Cervical Squamous Cell Carcinoma (CESC), and KIRC, higher expression levels were observed in the early stages, whereas in the remaining 7 tumor types, higher expression were linked to more advanced stages Statistical analysis confirmed the significance of these observations, with *p*-values < 0.05.

In the analysis of the relationship between tumor grading and HNRNPC expression, correlations were identified in 7 tumor types (Fig. 4M). Specifically, higher-grade tumors in PAAD, HNSC, Glioblastoma Multiforme (GBM), Lower-Grade Glioma (LGG), and LIHC exhibited elevated HNRNPC expression levels, while in KIPAN and KIRC, higher expression levels were associated with lower-grade tumors. The results indicate a possible involvement of HNRNPC in tumor prognosis.

Furthermore, ROC curve evaluation revealed that HNRNPC displayed substantial diagnostic precision (AUC > 0.8) for identifying 11 different cancers, including LIHC, LUAD, and KIHC (Fig. 4N–X). These results highlight the potential efficacy of HNRNPC as a diagnostic biomolecular marker across various cancer types.

### Association of HNRNPC with immune infiltration and immune checkpoint mechanisms

**Figure 5:**
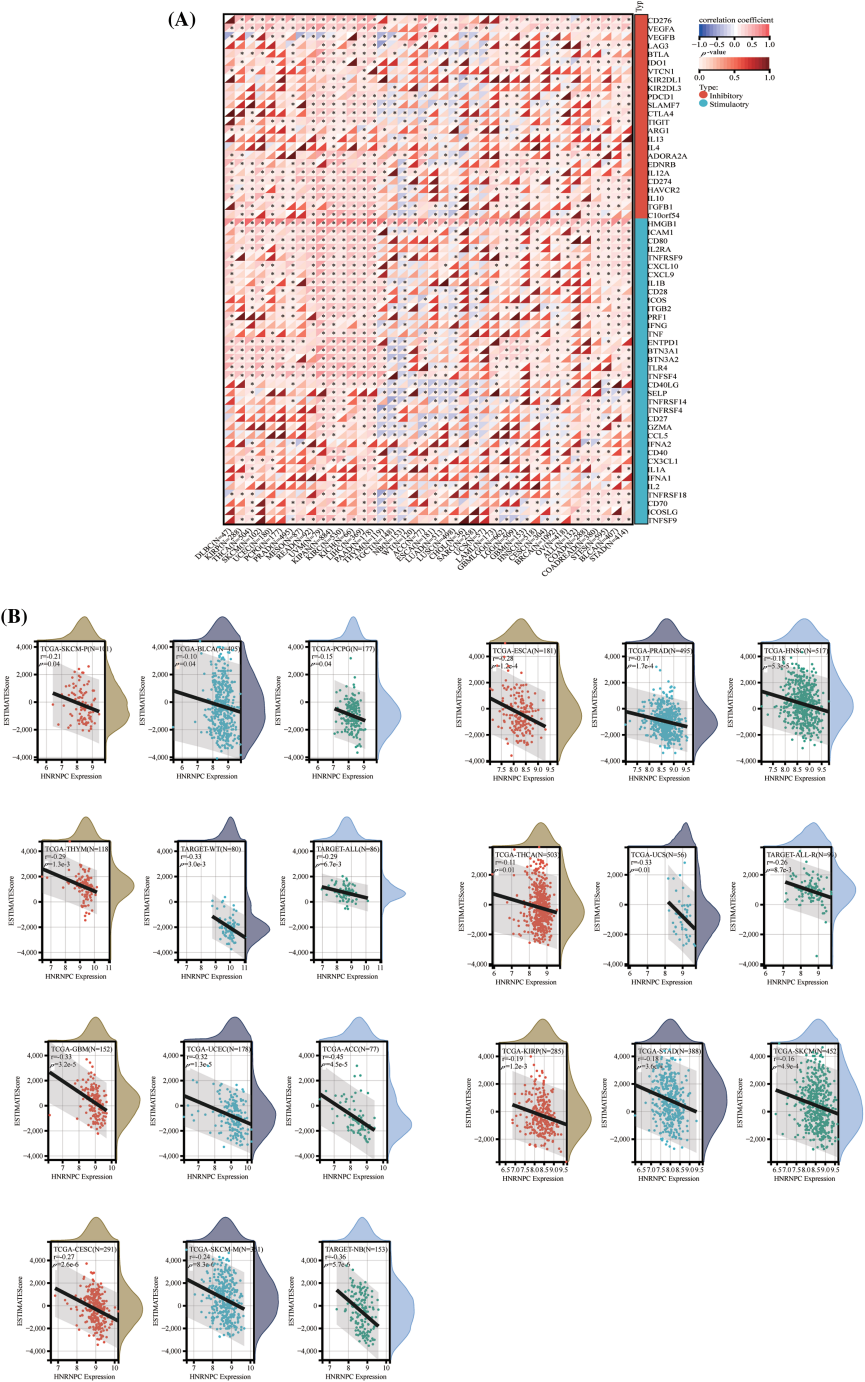
The correlation between HNRNPC expression and immune infiltration, alongside immune checkpoint markers. (A) The association between HNRNPC expression and genes associated with immune checkpoints. (B) The association between HNRNPC expression and EstimateScore. *p < 0.05.

To explore the impact of HNRNPC on the tumor microenvironment (TME), we examined the connection between HNRNPC levels and immune infiltration levels across various cancer types (Fig. 5A). Our analysis identified a noteworthy connection between HNRNPC and immune infiltration in 25 distinct cancer types, with positive correlations observed specifically in KIPAN and KIRC, while negative correlations were observed in the remaining 23 types of cancer (Fig. 5B). Similarly, the association between HNRNPC expression and three immune scores (ImmuneScore, EstimateScore, and StromalScore) displayed a consistent trend, with HNRNPC being negatively correlated with immune scores in most tumors. However, positive correlations were found in renal clear cell carcinoma and low-grade glioma (Fig. S2). These findings suggest that HNRNPC may play a crucial role in immune regulation within the tumor microenvironment across multiple malignancies. Notably, the observed negative correlation in a few tumors may be related to the heterogeneity among different tumors and the m6A modification effects of HNRNPC [37].

Furthermore, we investigated the association between HNRNPC expression and 60 genes involved in immune checkpoint pathways across various cancer types (Fig. 5A). HNRNPC exhibited significant positive correlations with immune inhibitory and immune stimulatory genes in the majority of tumors analyzed, indicating its potential involvement in regulating immune checkpoint mechanisms.

These observations highlight the intricate interplay between HNRNPC expression and tumor immunity, warranting further investigation into the underlying mechanisms driving these associations.

### Interaction of HNRNPC with immune cells

**Figure 6:**
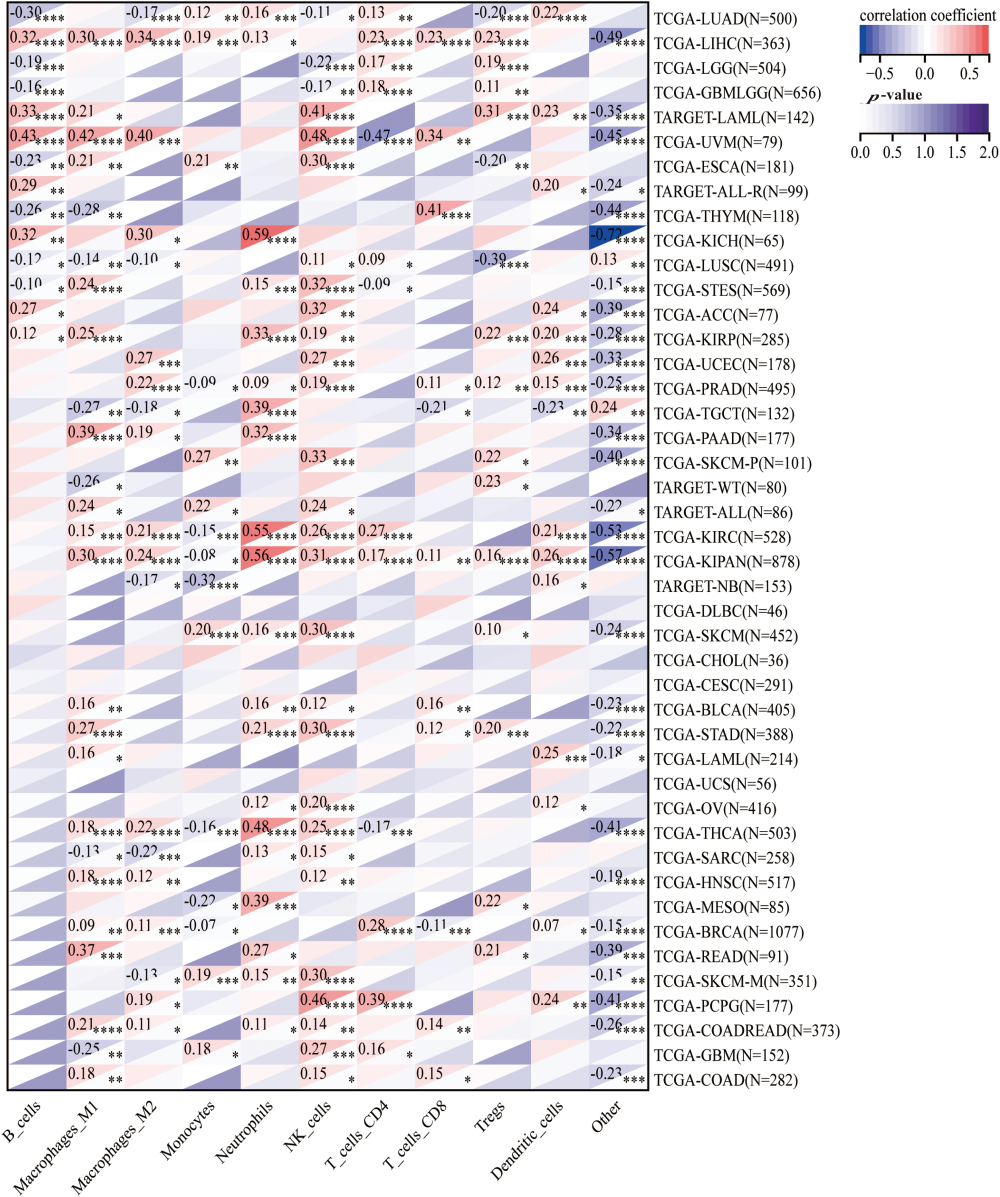
The association of HNRNPC with immune infiltrating cells. Unveiling the association of HNRNPC with immune infiltration levels in cancer through QUANTISEQ algorithm analysis. *p < 0.05; **p < 0.01; ***p < 0.001; ****p < 0.0001.

To explore the correlation between HNRNPC and tumor immunity, we employed four commonly used algorithms (QUANTISEQ, MCPcounter, EPIC, and TIMER) to evaluate immune scores across different tumors and examine the association between HNRNPC expression and immune cell levels. Our examination revealed a significant impact of HNRNPC expression on immune cell infiltration levels across various cancers, with natural killer cells, macrophages, and neutrophils exhibiting significant susceptibility (Figs. 6 and S3).

Specifically, we noted a notable positive correlation between HNRNPC expression and most immune cell populations across diverse malignancies, proposing a probable involvement of HNRNPC in sculpting the tumor immune microenvironment. These findings underscore the necessity for further investigation into the mechanisms governing the interaction between HNRNPC and immune cells within the context of cancer.

### Association of HNRNPC with immunomodulatory genes

**Figure 7:**
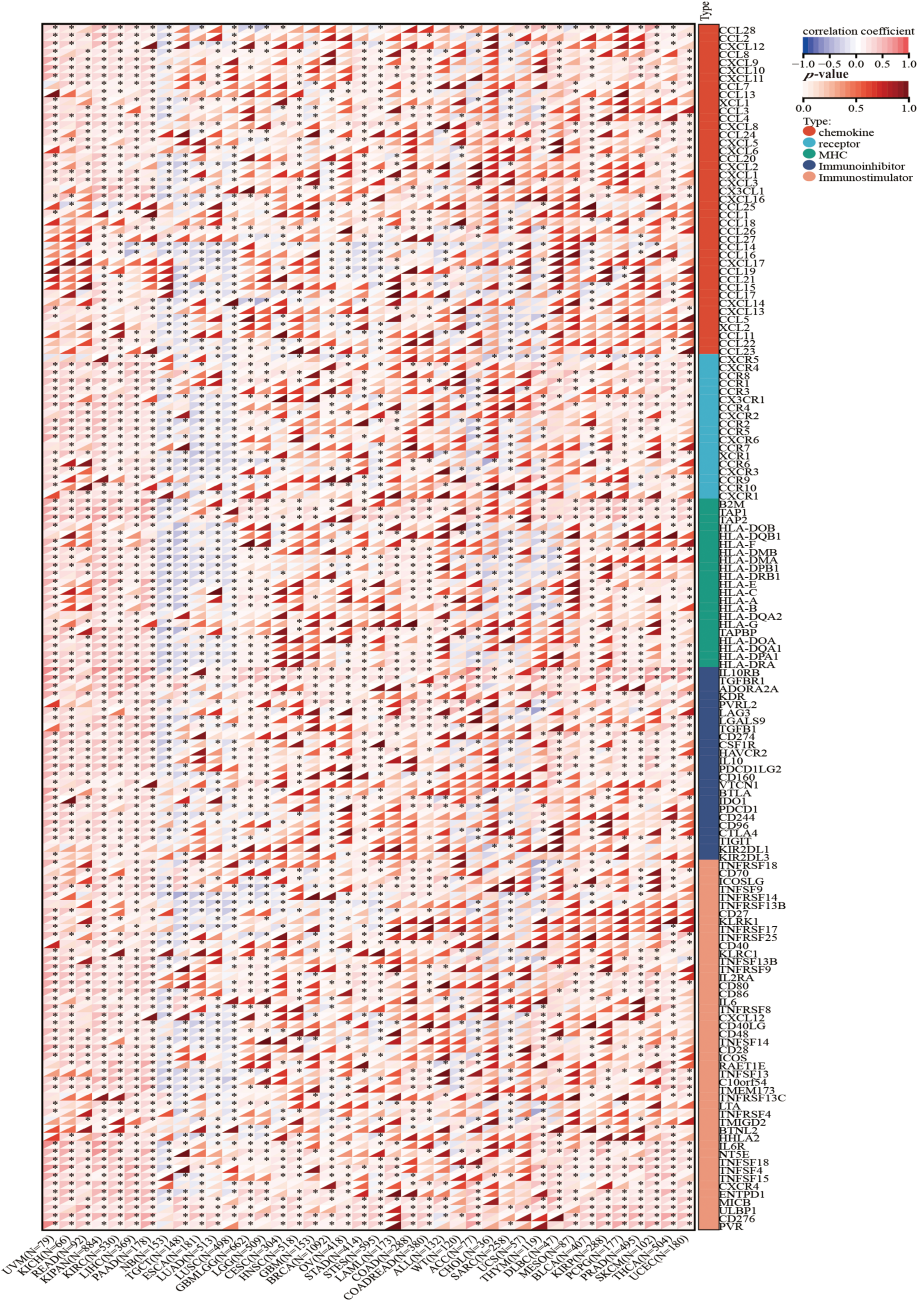
The correlation between HNRNPC and immune regulatory genes. HNRNPC is tightly associated with chemokines, receptors, MHC molecules, immunoinhibitors, and immunostimulators. *p < 0.05.

Apart from evaluating the relationship between HNRNPC levels and immune cell infiltration, we compared HNRNPC expression with common immunomodulatory-related gene families, encompassing chemokines, major histocompatibility complex (MHC), and chemokine receptors, across various tumor types. Our investigation unveiled a favorable association between HNRNPC expression and most genes belonging to these immunomodulatory gene families in the majority of tumors analyzed (except for Neuroblastoma (NB), Testicular Germ Cell Tumor (TGCT), Esophageal Carcinoma (ESCA), LUAD, and LUSC) (Fig. 7).

This finding suggests that HNRNPC expression may promote the formation of the immune microenvironment in most cancer cells by modulating the manifestation of immunomodulatory genes. Further exploration of the mechanisms underlying these correlations and their implications for tumor immunology and therapy is warranted.

### Prediction for treatment response with HNRNPC-mediated ICIS

Research has highlighted the importance of elements, for example, tumor mutation burden (TMB), microsatellite instability (MSI), tumor purity, and stemness score in predicting treatment response to immune checkpoint inhibitors (ICIs) and understanding tumor biology [38,39]. In this research, we investigated the association between HNRNPC expression and these tumor-related factors across diverse cancer types.

**Figure 8:**
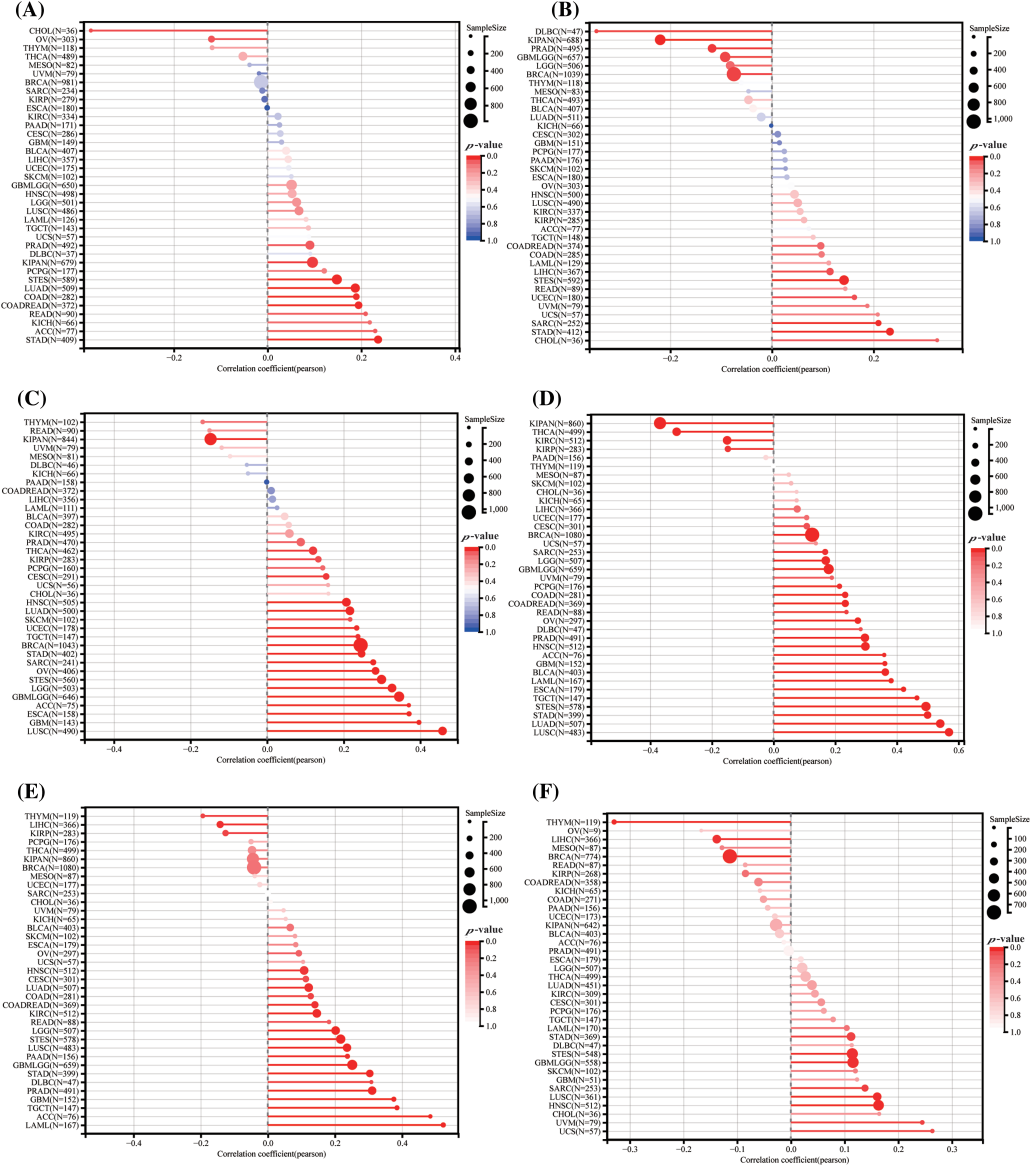
Forecasting the reaction to ICIS treatment through HNRNPC: (A) The association between HNRNPC levels and TMB (tumor mutation burden). (B) Correlation analysis between HNRNPC levels and MSI (microsatellite instability). (C) Association analysis of tumor purity. (D–F) Association analysis among HNRNPC levels and the stemness scores under EREG, RNASS, and DNASS.

Our investigation demonstrated a predominantly positive correlation between HNRNPC expression and both TMB and MSI across different cancer types. For instance, in LUAD, Colon Adenocarcinoma (COAD), READ, KICH, Adrenocortical Carcinoma (ACC), and Stomach Adenocarcinoma (STAD), HNRNPC levels demonstrated a notable association with TMB, while among LIHC, Small Cell Esophageal Cancer (STES), READ, Uterine *Corpus* Endometrial Carcinoma (UCEC), Uveal Melanoma (UVM), Uterine Carcinosarcoma (UCS), Sarcoma (SARC), STAD, and Cholangiocarcinoma (CHOL), HNRNPC expression exhibited a direct positive association with MSI. However, in some tumors, HNRNPC levels showed a negative correlation with TMB or MSI (Fig. 8A,B).

Additionally, we noted a positive correlation between HNRNPC expression and tumor purity in the majority of cancer types, consistent with the trends observed with TMB and MSI (Fig. 8C) [40]. Furthermore, our investigation disclosed a notable association between HNRNPC levels and stemness score in particular cancers, proposing their conceivable involvement in regulating drug resistance and tumor growth. (Fig. 8D–F) [41].

These findings underscore the complex interplay between HNRNPC expression and tumor biology, emphasizing its promise as a potential biomarker for predicting response to ICI therapy and its implications for personalized cancer treatment strategies.

### HNRNPC function as a biomarker in HCC

**Figure 9:**
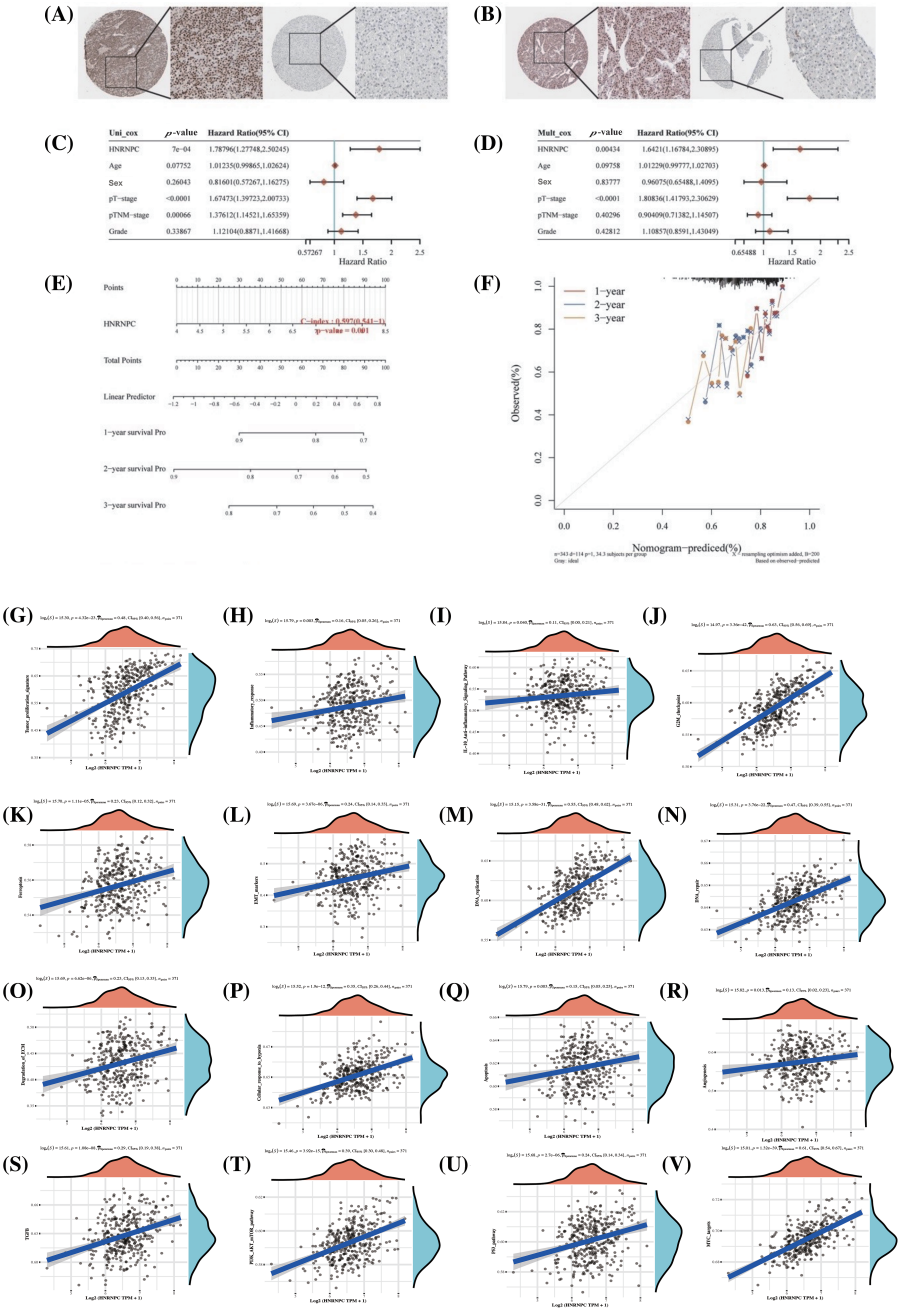
Assessment of HNRNPC’s clinical relevance in hepatocellular carcinoma. (A, B) Immunohistochemical staining of HNRNPC in liver tumor tissue and normal liver tissue. (C, D) Assessment of HNRNPC’s prognostic significance in liver cancer using both univariate and multivariate Cox analysis. (E) Creation of a nomogram grounded on HNRNPC expression. (F) Calibration analysis plot for the nomogram. (G–V) Correlation analysis of GSVA pathways with HNRNPC expression.

We validated the distinct expression patterns of HNRNPC in liver cancer compared to normal liver tissue through immunohistochemistry data from the HPA database (Fig. 9A,B). Consistent with previous findings, HNRNPC was significantly overexpressed in liver cancer. Survival analysis revealed robust associations between HNRNPC expression levels and disease-specific survival (DSS), overall survival (OS), progression-free survival (PFS), and disease-free survival (DFS) in patients with liver cancer (*p* = 3.2e-4, *p* = 6.5e-3, *p* = 1.1e-3, and *p* = 0.02, respectively). These associations were also closely interconnected with evaluations of TMB, MSI, and stemness scores.

Further analysis through univariate and multivariate Cox regression exposed that HNRNPC may function as a standalone prognostic indicator in liver cancer (Fig. 9C,D). Additionally, we developed a prognostic scoring system based on HNRNPC expression and pathological staging, enhancing the accuracy of prognostic evaluations in clinical settings (Fig. 9E). The model’s predictive accuracy was assessed using calibration curves, demonstrating its reliability in forecasting the prognosis of liver cancer patients over 1, 2, and 3-year intervals (Fig. 9F).

In order to further comprehend the physiological functions and underlying mechanisms associated with HNRNPC in HCC, we investigated its relationship with gene expression and pathway scores. Our investigation unveiled significant connections between HNRNPC levels and multiple pathways implicated in tumor progression, including angiogenesis, apoptosis, cellular response to hypoxia, extracellular matrix (ECM) degradation, DNA repair, DNA replication, epithelial-mesenchymal transition (EMT), ferroptosis, G2/M checkpoint, IL-10 anti-inflammatory response, immune response, and tumor proliferation (Fig. 9G–R). Furthermore, GSVA analysis suggested that HNRNPC may influence malignant proliferation in liver cancer through its impact on the MYC, TGFB, P53, and PI3K-AKT-mTOR pathways (Fig. 9S–V).

### HNRNPC knockdown significantly inhibits liver cancer cell proliferation, invasion and migration

Building upon the aforementioned findings, our investigation unveiled a significant increase in HNRNPC expression across a spectrum of tumor types, notably hepatocellular carcinoma (HCC), when compared to normal controls. Furthermore, a strong association was noted between HNRNPC expression levels and four pivotal survival intervals: overall survival (OS), disease-specific survival (DSS), disease-free survival (DFS), and progression-free survival (PFS).

**Figure 10:**
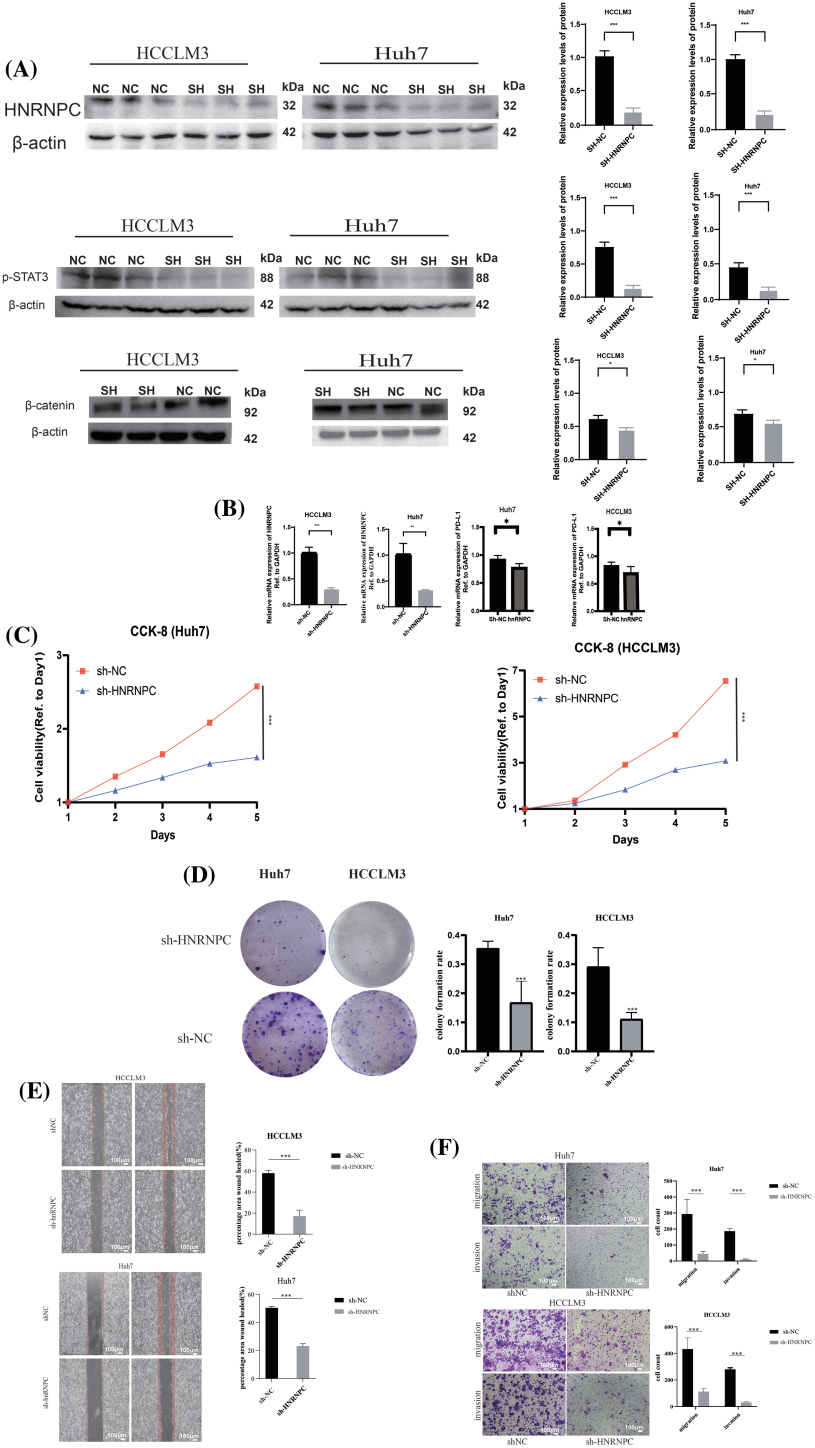
The impact of suppressing HNRNPC expression on the proliferation, invasion, and metastatic potential of liver cancer cells (A) Western blot: HNRNPC, p-STAT3 and β-catenin protein level in Huh7 and HCCLM3. (B) Histograms of HNRNPC and PD-L1 mRNA level in negative control and sh-HNRNPC Huh7 and HCCLM3 cells. (C) CCK8 assay showed that suppressing HNRNPC expression inhibited the proliferation of Huh7 and LM3 cells. (D) Representative images of colony formation in Huh7 and HCCLM3, histograms of colony formation rate in two cell lines. (E) Representative images of wound-healing assay in Huh7 and HCCLM3. (F) Representative images showing migration and invasion in Huh7 and HCCLM3 cells. *p < 0.05; **p < 0.01; ***p < 0.001.

To mechanistically verify the impact of HNRNPC on tumor development, we used liver cancer cell lines as experimental models. Specifically, we chose two established liver cancer cell lines, Huh7 and HCCLM3, for functional validation. By employing lentiviral vectors to silence the HNRNPC sequence in these cell lines, we then selected stable expressing cells. The effectiveness of HNRNPC knockdown was confirmed through Western blotting (WB) and reverse transcription polymerase chain reaction (RT-PCR) analyses. Additionally, WB experiments validated the impact of HNRNPC on STAT3 and β-catenin, showing that HNRNPC knockdown inhibits the STAT3 and β-catenin pathways, consistent with previous studies. Furthermore, PCR analysis revealed a downregulation of PD-L1 gene expression following HNRNPC knockdown (Fig. 10A,B).

Subsequently, cellular viability assays using CCK8 and colony formation assays were conducted to assess the proliferative capacity of Huh7 and HCCLM3 cells upon HNRNPC downregulation (Fig. 10C,D). Notably, the results showed a significant suppression of cell proliferation following HNRNPC knockdown.

Furthermore, invasion and migration experiments, including wound-healing tests, were conducted to evaluate the impact of HNRNPC knockdown on the invasive and migratory abilities of Huh7 and HCCLM3 cells (Fig. 10E,F). Remarkably, our findings revealed a significant inhibition of both invasion and migration in Huh7 and HCCLM3 cells upon HNRNPC knockdown.

These experimental results underscore the critical role of HNRNPC in the progression of hepatocellular carcinoma and highlight its potential as a therapeutic target for liver cancer, particularly as a target for immunotherapy.

### Validation of the impact of HNRNPC on hepatocellular carcinoma was conducted through in vivo experiments

**Figure 11:**
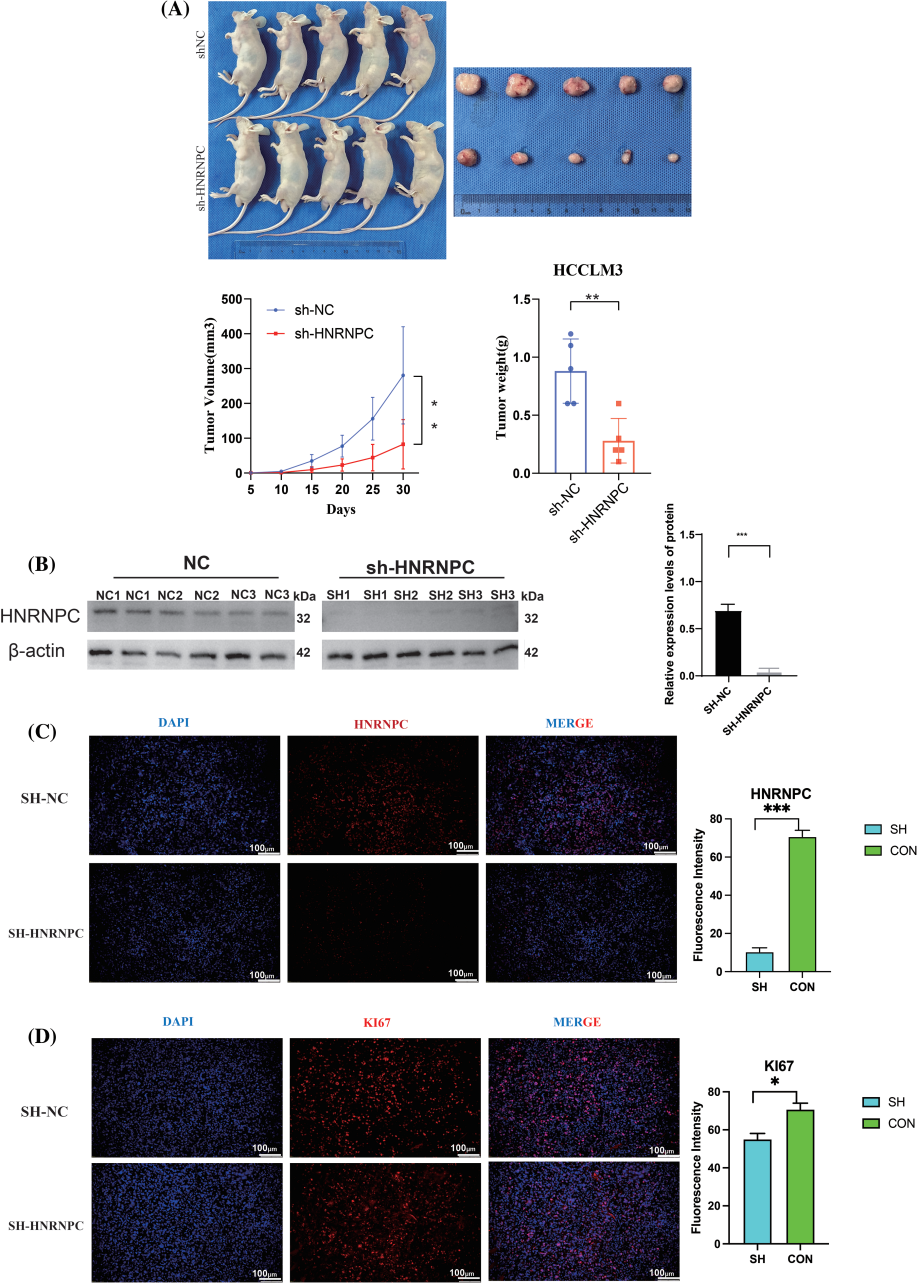
Revalidation of the effect of HNRNPC on liver cancer *in vivo*. (A) Images of subcutaneous tumor formation in nude mice, n = 5, the volume of the tumor was recorded within 30 days, then tumors were taken. (B) Western blolt detection of HNRNPC protein levels in subcutaneous tumor tissue. (C) Representative images of immunofluorescence in negative control and sh-HNRNPC group. (D) Immunofluorescence: expression of ki67 in control cohort and sh-HNRNPC cohort. *p < 0.05; **p < 0.01; ***p < 0.001.

Subcutaneous implantation experiments were conducted using treated HCCLM3 cells and negative control HCCLM3 cells. Upon growth of the tumor tissues, the subcutaneous tumors were harvested for evaluation of tumor formation in nude mice, revealing significant suppression of tumorigenic potential following HNRNPC gene knockdown in HCCLM3 cells (Fig. 11A). After detecting the HNRNPC protein levels in subcutaneous tumor tissues using Western blot, it was found that the HNRNPC protein amount in the knockdown group was significantly lower than that in the control group (Fig. 11B). Further immunofluorescence staining analysis of the extracted subcutaneous tumors confirmed successful HNRNPC silencing (Fig. 11C), indicated by reduced Ki67 fluorescence signal, a proliferation index marker, after HNRNPC gene knockdown (Fig. 11D).

### Knocking down HNRNPC can influence the immune microenvironment and malignancy of liver cancer tissues in vivo

**Figure 12:**
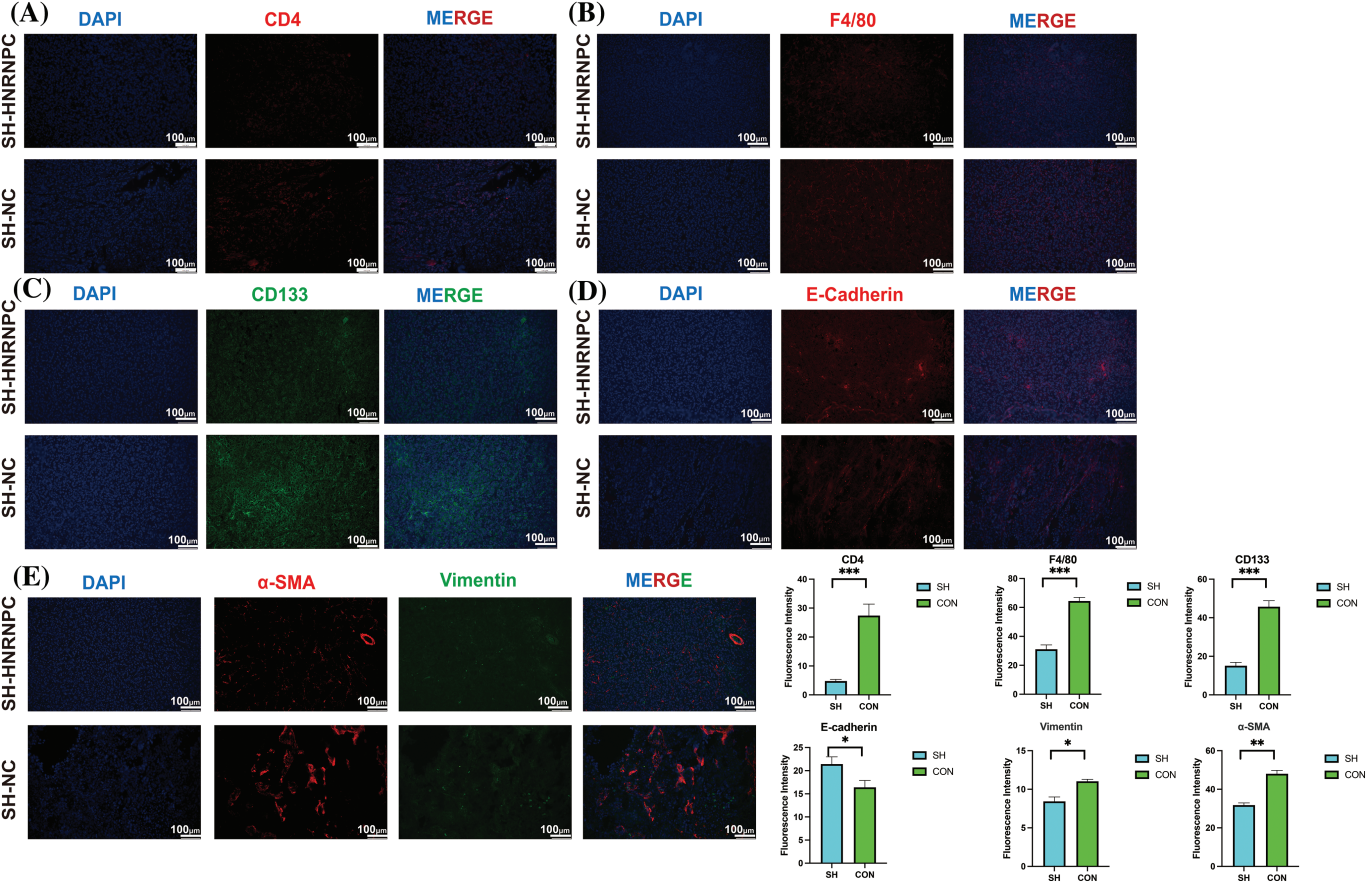
Immuno-fluorescence staining confirmed a close correlation between HNRNPC and tumor immunity as well as malignancy. (A, B) Immunofluorescence: expression of CD4 and F4/80 in control group and sh-HRNPC group. (C–E) Immunofluorescence: expression of CD133, E-cadherin, α-SMA and Vimentin in control group and sh-HRNPC group. *p < 0.05; **p < 0.01; ***p < 0.001.

Through further immunofluorescence analysis of subcutaneous tumor tissues from liver cancer cells, we validated findings that HNRNPC expression is closely associated with levels of tumor-infiltrating immune cells. We observed that in tumor tissues with HNRNPC knockdown, we observed a significant reduction in the expression of CD4 and F4/80 compared to the control group with intact HNRNPC expression (Fig. 12A,B). Additionally, we investigated the relationship between HNRNPC and malignant processes in tumors, such as epithelial-mesenchymal transition (EMT) and tumor stemness. Immunofluorescence staining revealed that in tumors with HNRNPC knockdown, the expression levels of CD133, α-SMA, and Vimentin were significantly decreased compared to the control group, while E-cadherin expression was significantly higher than in tumors where HNRNPC was not knocked down (Fig. 12C–E). These findings further confirm that HNRNPC can affect the tumor microenvironment and is closely linked to tumor malignancy.

## Discussion

Cancer research occupies a central position in the medical domain. It focuses on hallmark features such as the maintenance of proliferative signaling, avoidance of growth suppression, resistance to apoptosis, achieving replicative immortality, gaining access to blood vessels, triggering invasion and metastasis, reconfiguring cellular metabolism, and evading immune destruction [8]. HNRNPC, an RNA-binding protein from the hnRNP family, regulates multiple aspects of RNA metabolism, encompassing alternative splicing, mRNA stability, and translation. Dysregulation of HNRNPC emerges as pivotal across various cancer types, positioning it as a putative pan-cancer gene implicated in diverse malignancies. In this study, we embarked on an investigation to identify potential biomarkers for broad-spectrum cancer diagnosis by scrutinizing gene expression alterations across 33 cancer-related datasets sourced from TCGA and GTEx platforms.

Our pan-cancer expression analysis unveiled significant upregulation of HNRNPC in nearly all cancer lineages, with the exceptions of KIRP, KIPAN, KIRC, OV, and PCPG. Particularly notable overexpression was observed in certain cancers, including GBM, BRCA, LUSC, and LIHC. Subsequent Cox regression and Kaplan–Meier survival analyses provided partial confirmation of HNRNPC’s potential as a reliable biomarker. Comparison with survival outcomes underscored significant associations of HNRNPC with OS, DSS, DFS, and PFS across 11 cancer types, including LUAD, LIHC, LAML, ACC, KIRP, KIPAN, SARC, HNSC, KICH, and PAAD. This highlights the imperative to elucidate its underlying mechanism.

Previous investigations by Lian et al. and Cheng et al. have shed light on the role of HNRNPC in breast cancer and prostate cancer, respectively, unveiling its involvement in promoting tumor progression [19,24]. Moreover, numerous studies across various cancers, such as oral squamous cell carcinoma [42], NSCLC [43,44], esophageal squamous cell carcinoma [45,46], clear cell renal cell carcinoma [47], chronic lymphocytic leukemia [48], glioma cells [49], and gastric cancer [50], have underscored the significant contribution of HNRNPC to tumor progression.

The tumor microenvironment (TME), comprising diverse immune cell populations, cancer-associated fibroblasts, stromal cells, and endothelial cells, exerts a critical influence on cancer pathogenesis [51]. Notably, studies by Cheng et al. have elucidated HNRNPC’s role in inhibiting tumor immunity and promoting immune evasion in prostate cancer and breast cancer, respectively [24]. However, further analysis is warranted to explore other immune features, including immune regulatory genes, which prompted our study’s emphasis on investigating the association between HNRNPC and the immune microenvironment [52].

Our investigation unveiled a close association between HNRNPC levels and immune infiltration levels, as evidenced by significant correlations with immune scores across multiple cancer types. Notably, a predominantly negative correlation was noted among HNRNPC levels and immune infiltration levels, particularly notable in certain cancers like WT, TGCT, and GBM. This underscores the potential significance of HNRNPC in these tumors and highlights avenues for future research elucidating the underlying mechanisms.

Additionally, our analysis unveiled a positive link between HNRNPC levels and key factors influencing the suitability of immune checkpoint therapy, including TMB, MSI, and tumor purity, across most cancers. These findings hint at the potential impact of HNRNPC on immunotherapeutic efficacy and tumor progression, warranting further investigation into its mechanistic implications.

Moreover, the pronounced positive association observed between HNRNPC and ICI-related genes in various cancers suggests its potential role in modulating immune checkpoint pathways and evading immune surveillance. The observed correlations with immune cell infiltration, immune regulatory genes, and pathway scoring further underscore HNRNPC’s intimate association with tumor immunity and its therapeutic target’s potential.

In the context of hepatocellular carcinoma (HCC), our analysis identified HNRNPC as an independent prognostic marker, with meaningful correlations observed between HNRNPC expression and various pathways implicated in tumor progression. Experimental validation through *in vitro* and *in vivo* models further corroborated these findings. The influence of HNRNPC on the progression of HCC, underscoring its prospective value as both a therapeutic option and prognostic marker for HCC and various other cancers.

Undeniably, our study had several limitations. We did not conduct comprehensive transcriptomic and proteomic analyses, which limited our detailed exploration of HNRNPC’s functions in tumor cells. We will pursue more in-depth research on this in future studies. Additionally, the impact of m6A modifications on HNRNPC has been receiving increasing attention, and we will also investigate this in future research. This could potentially explain why the effect of HNRNPC on the immune microenvironment differs in a few tumors compared to most tumors.

## Conclusion

In summary, our pan-cancer analysis elucidates the expression profile disparity of HNRNPC across a spectrum of malignancies, its prognostic implications, and its intricate involvement in tumor immunity. The findings underscore HNRNPC’s potential as a potential focus of therapy and a predictive indicator of outcomes in diverse cancers, warranting further exploration to unravel its mechanistic intricacies and therapeutic potential.

## Supplementary Materials

Figure S1 HNRNPC has a close association with overall survival (OS) in various tumors. **(A-H)**: Kaplan-Meier (**KM**): survival analysis further confirmed the association between HNRNPC and OS in HNSC, KIPAN, KIRC, KIRP, LAML, SRAC, LUAD, and LIHC cancer types.

Figure S2 The close correlation between HNRNPC expression and immune infiltration **(A)**: The correlation between HNRNPC expression and StromalScore (**B**): The correlation between HNRNPC expression and StromalScore.

Figure S3 The correlation of HNRNPC with the level of immune infiltrating cells. (**A-C**): Revealing the correlation between HNRNPC and immune infiltration level in cancer through MCPcounter, EPIC and TIMER analysis.

## Data Availability

The datasets generated during and/or analyzed during the current study are available from the corresponding author on reasonable request.

## Supplementary Materials

The supplementary material is available online at https://doi.org/10.32604/or.2024.055866.
